# Histological Identification of* Propionibacterium acnes* in Nonpyogenic Degenerated Intervertebral Discs

**DOI:** 10.1155/2017/6192935

**Published:** 2017-03-19

**Authors:** Ye Yuan, Zezhu Zhou, Yucheng Jiao, Changwei Li, Yuehuan Zheng, Yazhou Lin, Jiaqi Xiao, Zhe Chen, Peng Cao

**Affiliations:** ^1^Department of Orthopedics, Ruijin Hospital, Shanghai Jiao Tong University School of Medicine, Shanghai, China; ^2^Shanghai Key Laboratory for Prevention and Treatment of Bone and Joint Diseases with Integrated Chinese-Western Medicine, Shanghai Institute of Traumatology and Orthopedics, Ruijin Hospital, Shanghai Jiao Tong University School of Medicine, Shanghai, China; ^3^Department of Orthopedic Surgery, Xinhua Hospital, Shanghai Jiao Tong University School of Medicine, Shanghai, China; ^4^Department of Orthopedics, Ruijin Hospital North, Shanghai Jiao Tong University School of Medicine, Shanghai, China; ^5^Department of Medical Microbiology and Parasitology, Shanghai Jiao Tong University School of Medicine, Shanghai, China

## Abstract

*Purpose*. Low-virulence anaerobic bacteria, especially the* Propionibacterium acnes (P. acnes)*, have been thought to be a new pathogeny for a series of disc diseases. However, until now, there has been no histological evidence to confirm this link. The purpose of this study was to confirm the presence of* P. acnes* in nonpyogenic intervertebral discs via histological observation.* Method*. Degenerated intervertebral discs were harvested from 76 patients with low back pain and/or sciatica but without any symptoms of discitis or spondylodiscitis. The samples were cultured under anaerobic conditions and then examined using 16S rDNA PCR to screen for* P. acnes*. Samples found to be positive for* P. acnes* were stained with hematoxylin-eosin (HE) and modified Brown-Brenn staining and observed under a microscope.* Results*. Here, 16 intervertebral discs were found to be positive for* P. acnes* via 16S rDNA PCR and the prevalence was 21.05% (16/76). Among them, 7 samples had visible microbes stained with HE and modified Brown-Brenn staining. Morphological examination showed the bacteria to be Gram-positive and rod-shaped, so they were considered* P. acnes*.* Conclusion*.* P. acnes* is capable of colonizing some degenerated intervertebral discs without causing discitis, and its presence could be further confirmed by histological evidence. Targeting these bacteria may be a promising therapy method for some disc diseases.

## 1. Introduction

Since the first isolation of low-virulence anaerobic bacteria, their pathological roles in nonpyogenic intervertebral discs have drawn an increasing amount of attention. Previous studies have demonstrated that the prevalence of low-virulence anaerobic bacterium ranged from 20% to 53% [1–7]. Among them,* P. acnes* has been found to account for as much as 84% of all isolated bacteria [7]. It is considered the predominant bacterium. Interestingly, infection with this bacterium has not been found to cause any pathological changes in discitis or spondylodiscitis.

However, epidemiological studies suggested that latent infection of the low-virulence anaerobic bacteria, especially the* P. acnes*, had a strong correlation with disc degeneration, Modic changes, sciatica, and low back pain [4, 7–9]. For example, some clinical studies have demonstrated that antibiotic therapy targeting the bacteria significantly reduced the low back pain and Modic changes in patients [9, 10]. In addition, inoculation of* P. acnes* into normal intervertebral discs of rats or rabbits induced severe disc degeneration and Modic changes [11, 12]. For this reason,* P. acnes* is thought to be involved in a previously undiscovered pathogeny for series of disc diseases.

Nevertheless, some studies have strongly repudiated the idea of a pathological role of* P. acnes* in intervertebral discs. One rationale is that the* P. acnes* isolated in these cases may have been contamination rather than original growth because* P. acnes* is commensal flora existing on skin and it may readily contaminate the intervertebral discs during tissue harvesting [13, 14]. Another point was that some studies did not find any bacteria at all [15–17]. For example, one study covering more than 300 samples found no original growth of* P. acnes* in any intervertebral discs [15].

Generally, the controversy is attributable to the lack of histological evidence. The gold standard diagnostic method, histological identification of bacteria, would not only resolve the question of contamination but also provide undoubtable evidence of its presence in intervertebral discs. For this reason, the primary purpose of this study was to confirm the presence of* P. acnes* in nonpyogenic intervertebral discs via histological examination. The prevalence of* P. acnes* was also investigated here. This was the first study to confirm the presence of* P. acnes* via histological observation, and it provides critical evidence that may dispel this controversy.

## 2. Methods and Materials

### 2.1. Patients and Tissue Harvesting

A total of 76 patients were included in this study from September 2013 to October 2015. The patients underwent discectomy at single-level lumbar spine due to disc degeneration associated with low back pain and/or sciatica. Patients underwent conservative treatment before surgery. The patients who received antibiotics within the month preceding surgery were not included in this study. Among included patients, the average age was 55.30 ± 14.59 years, and 39 patients were male and 37 patients were female. The levels of surgery were as follows: 2 at L2~3, 8 at L3~4, 41 at L4~5, and 25 at L5~S1. The study was approved by the Institutional Review Board of Shanghai Ruijin Hospital and informed consent forms were provided by every patient.

Intervertebral discs were harvested using a routine approach, posterior discectomy with or without interbody fusion. A stringent antiseptic sterile protocol described in a previous study [8] was used to prevent contamination to the greatest extent possible. Briefly, the skin of the operation field was disinfected and sterilized 3 times with povidone iodine and then allowed to dry. Then a 3M Ioban 2 Antimicrobial Incise Drape (3M Health Care, St. Paul, MN, USA) was used to cover the surgical field, which also helped prevent bacterial contamination from dermis or subdermis. In addition, before the discectomy of intervertebral discs, the wound was irrigated twice using sterilized water. Next, the specimen was handled exclusively with fresh instruments to minimize contamination. Finally, some muscle samples adjacent to the intervertebral discs were collected after discectomy. These served as markers of contamination during surgery. Some of the harvested intervertebral discs along with the contamination marker muscle were used for bacterial isolation and identification. Samples taken from the remaining tissues were used for histological examination.

### 2.2. Tissue Culture and PCR Examination

As in a previous study, all of the samples of intervertebral discs and muscles were immersed in 9 mL tryptone soy broth (TSB) enriched with 10% bovine serum [8]. Tubes containing enriched TSB were incubated in an anaerobic glove box instrument (80% N_2_, 10% CO_2_, 10% H_2_) at 37°C for 14 days. To prevent contamination during culture, a pure culture medium without tissue was incubated and examined under the same conditions, serving as a blank control.

At the end of the follow-up period, all broth was examined via polymerase chain reaction (PCR) for amplification of 16S rDNA gene. In accordance with the method described by Albert et al. [4] and our previous study [8], specific primers were designed to amplify a 600 bp region of* P. acnes*. The forward primer was 5′-GGGTTGTAAACCGCTTTCGCCT-3′, and the reverse primer was 5′-GGCACACCCATCTCTGAGCAC-3′. A boil method was used to extract template DNA from culture broth. A positive control (with DNA from standard strain of* P. acnes* provided by Guangzhou Type Culture Collection, ATCC #6919) and a negative control (sterile water as template) were included. The PCR was performed as a mixture of 20 *μ*L volume containing 0.8 *μ*L of each primer, 2 *μ*L of 2.5 mM MgCl2, 1.6 *μ*L of dNTP, 2 *μ*L of 10x Ex Taq Buffer, 0.2 *μ*L of 5 U/*μ*L Ex Taq, 2 *μ*L of template DNA, and 10.6 *μ*L of sterilized distilled water (Takara RR01AM, Japan). The PCR conditions were as follows: 94°C preheating for 4 min, 94°C denaturation for 45 s, 35 cycles of 94°C denaturation for 45 s, 57°C annealing for 45 s, 72°C extension for 1 min, and a final extension for 7 min at 72°C. Then the amplified product (5 *μ*L) was collected and run on a 1% agarose gel containing 1 *μ*g/ml of ethidium bromide. Electrophoresis was performed in 1x TAE (40 mM Tris-AC, 1 mM EDTA) glacial acetic acid buffer at 120 V. DNA bands were visualized using UV light photography.

### 2.3. Bacterial Isolation and Gram-Staining

At the end of culture, the broth that had been identified as* P. acnes*-positive using 16S rDNA was transferred onto Columbia blood agar plate for further bacterial isolation and purification. Briefly, 1 mL of TSB was transferred onto the plates and incubated for 48 h at 37°C under anaerobic conditions (80% N_2_, 10% CO_2_, and 10% H_2_). Then, a single colony was selected and transferred onto Columbia blood agar plates to culture for another 24 h.

For Gram-staining identification, a single colony was selected, transferred into slides, and fixed. The samples were stained with crystal violet for 1 min and washed with water. Then the samples were stained with Gram's iodine for 1 min and washed with water again. The samples were then decolorized with 95% alcohol for 20 s and washed. Finally, the samples were stained with safranin for 1 min and rinsed with water. After staining, each slide was dried in the air and mounted with a coverslip.

### 2.4. Histological Examination of HE and Modified Brown-Breen Staining

Intervertebral discs harvested from patients were fixed in 4% formaldehyde for 24 h and then processed with routine paraffin embedding and sectioned at 5 *μ*m. Routine HE and modified Brown-Brenn staining were performed for each slide. For modified Brown-Brenn staining, the sections were first deparaffinized and rehydrated and then stained with crystal violet for 2 min and then with Lugol's iodine for 2 min. After that, the sections were dipped into acetone for 30 s and washed in water. Then 0.25% basic fuchsin was dropped onto the sections, which were incubated for 5 min. The sections were decolorized with Gallego's solution (0.5 mL glacial acetic acid, 1 mL 3.7% formalin, and 50 mL distilled water) for 5 min and then quickly washed twice with acetone. Then the sections were stained with picric acid-acetone mixture (5 mL saturated picric acid and 45 mL acetone) for 30 s and decolorized 3 times with acetone. Finally, the sections were hyalinized with xylene and mounted with coverslips.

After modified Brown-Brenn staining, the Gram-positive bacteria were stained blue and the Gram-negative bacteria red. All stained samples were observed under a microscope with an oil immersion lens at a magnification of 630x (Axio, Carl Zeiss, Oberkochen, Germany) and digital images were captured at the same time. During the observation, 5 slides were selected for each patient and 10 randomly selected fields per slide were checked.

## 3. Results

### 3.1. The Prevalence of* P. acnes* in Intervertebral Discs

Of the 76 patients, 20 (20/76, 26.32%) were found to have* P. acnes* in intervertebral discs after anaerobic culture and 16S rDNA PCR examination. However, among the 20 patients, 16 patients (16/76, 21.05%) had* P. acnes *only in intervertebral discs and the culture results of adjacent muscle were negative. Another 4 patients had positive results for* P. acnes* in both intervertebral discs and adjacent muscles. The samples of adjacent muscles served as a marker of contamination, and positive results in the muscles indicated the presence of contamination associated with the incision during surgery. This suggests that the* P. acnes* isolated from these 4 patients may stem from contamination during tissue harvesting during surgery while the* P. acnes* isolated from the other 16 patients was original growth (Table 1).

**Figure 1:**
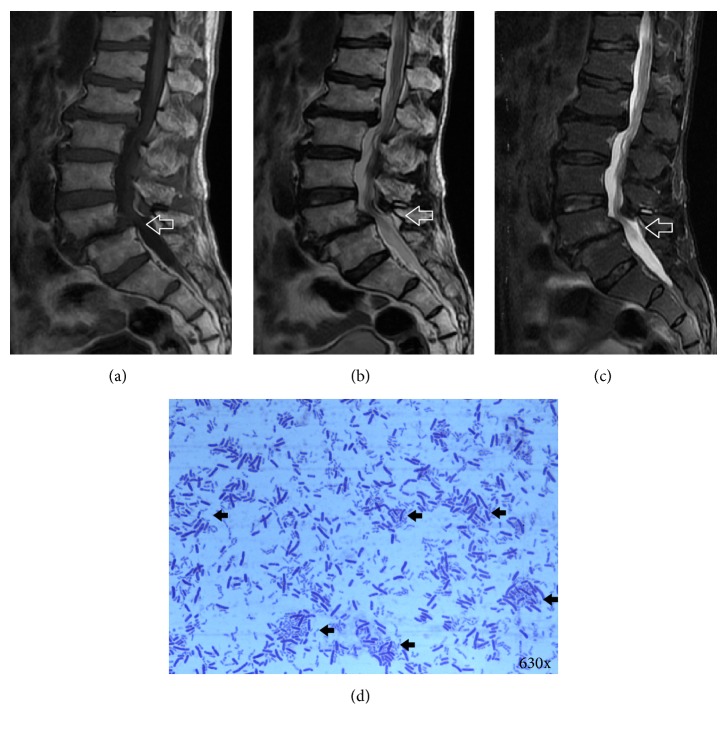
*P. acnes* identified and isolated from a nonpyogenic intervertebral disc. (a)–(c) A 68-year male patient (patient's ID: number 9) underwent discectomy due to severe low back pain and sciatica. The disc harvested from the patient at level of L4~5 was proved to be* P. acnes*-positive using anaerobic culture and 16S rDNA PCR. The signs of discitis, such as osteolytic changes of endplates or vertebrae, pyogenic changes of disc, and septic changes of vertebral surrounding soft tissues, were not seen in T1WI (a), T2WI (b), and STIR (c) of MRI (marked with white blank arrows). (d) Rod-shaped, Gram-positive bacterium was isolated and purified from the* P. acnes*-positive disc (marked with black arrows) and the purified bacterium was* P. acnes*.

In addition, the intervertebral discs of 3 patients (3/76, 3.95%) also showed bacterial growth in the broth during culture, but the results of 16S rDNA PCR suggested that they were not* P. acnes*. Finally, 53 patients (53/76, 69.74%) were absolutely bacteria-free in both discs and adjacent muscles (Table 1). The* P. acnes* isolated from the 16 patients was further purified on an agar plate and examined with Gram-staining. Rod-like, Gram-positive bacteria were found in each colony (Figure 1(d)).

### 3.2. Histological Identification of* P. acnes* in Intervertebral Discs

Of the 16 intervertebral discs identified as* P. acnes*-positive via 16S rDNA PCR, 15 samples were investigated further via histological examination (1 positive patient did not have enough tissue for histological examination). None of the patients had any symptoms of discitis before surgery, such as fever or chills. Upon laboratory examination, the white blood cell counts were within normal ranges and there were no indications of infection. Only different degrees of disc degeneration with or without Modic changes were observed in MRI. There were no signs of discitis in MRI, such as osteolytic changes in endplates or vertebrae, pyogenic changes in the discs, or septic changes in the vertebral area surrounding soft tissues. All of 15* P. acnes*-positive patients were diagnosed with nonpyogenic degenerated intervertebral discs (Figures 1(a)–1(c)).

**Figure 2:**
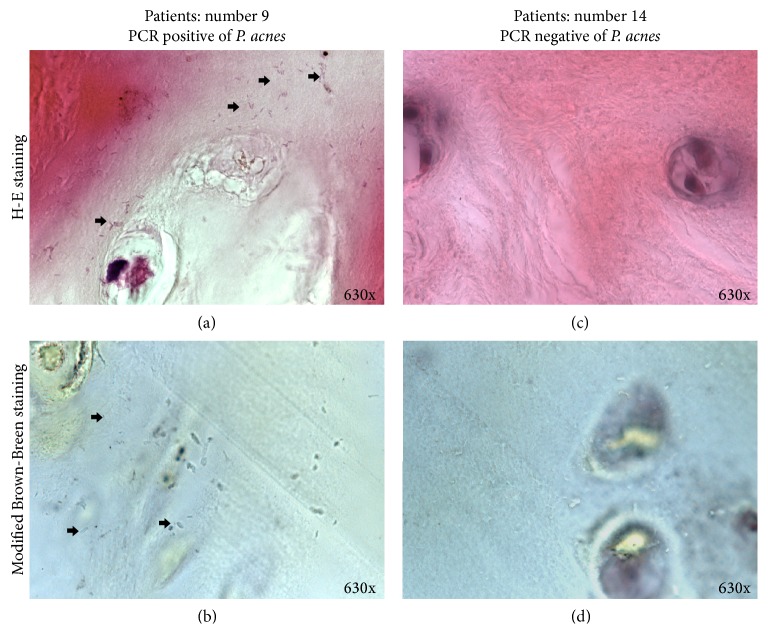
Histological evidence of* P. acnes* in nonpyogenic degenerated intervertebral disc. (a) HE staining revealed rod-shaped bacteria growing in cluster (marked with black arrows); (b) modified Brown-Brenn staining further revealed the bacteria as blue, indicating that they were Gram-positive (marked with black arrows). The intervertebral disc was harvested from the patient described in Figure 1, who was identified as* P. acnes*-positive using 16S r DNA PCR at the level of L4~L5 (patient's ID: number 9). (c)-(d) By contrast, no bacterium was found in either HE or modified Brown-Brenn staining in a* P. acnes*-negative intervertebral disc.

**Figure 3:**
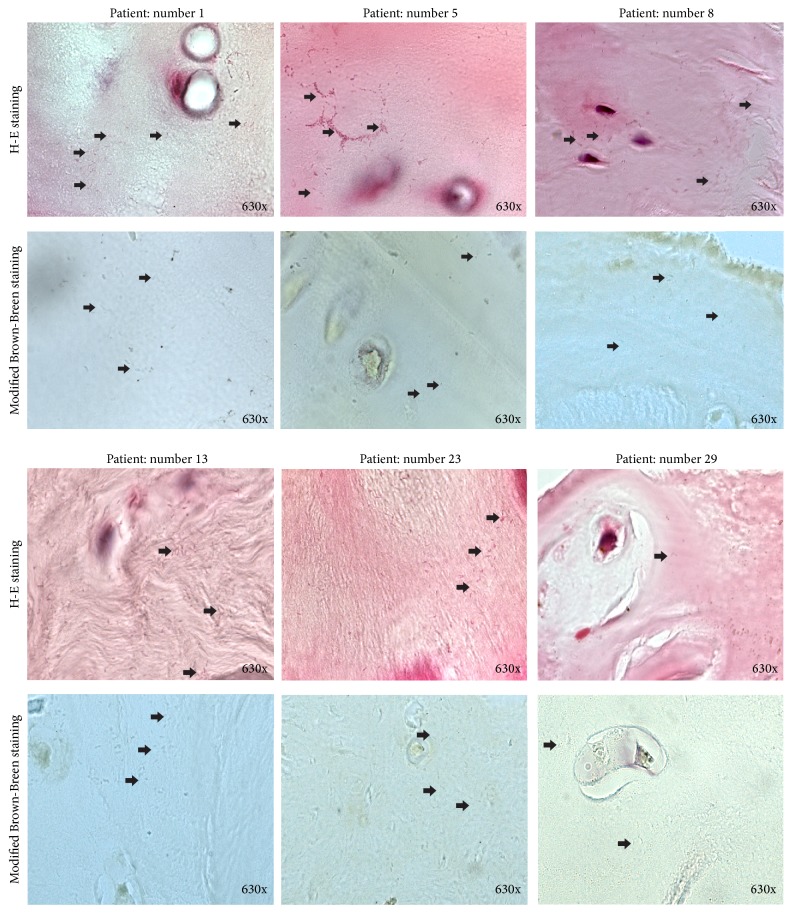
Histological images of other six* P. acnes*-positive intervertebral discs. Gram-positive and rod-shaped bacteria were marked with black arrows in each image.

Of the 15 samples, 7 samples had visible bacteria after HE and modified Brown-Brenn staining, accounting for 9.21% (7/76, Figures 2 and 3). These bacteria appeared rod-shaped, which was similar to the morphology of purified* P. acnes* (Figure 2(a)). In addition, modified Brown-Brenn staining turned the rod-shaped microbes blue, indicating that they were Gram-positive (Figure 2(b)). Considering that the 7 intervertebral discs had been identified as* P. acnes*-positive via 16S rDNA PCR examination, the observed rod-shaped, Gram-positive bacteria were here considered as* P. acnes*.

Some bacteria grew in clusters inside the intervertebral discs (Figures 2 and 3), further suggesting that the observed bacteria were original growth rather than contamination. A previous study found large aggregates of bacteria to be highly suggestive of bacterial infection and single bacterial cells suggested intraoperative contamination [13].

Here, 15 intervertebral discs found to be bacteria-free using 16S rDNA PCR were randomly selected and examined using the same histological methods. No bacteria were found using either HE or modified Brown-Brenn staining (Figures 2(c) and 2(d)).

## 4. Discussion

In this study, nonpyogenic degenerated intervertebral discs were confirmed to have latent* P. acnes* infections at a prevalence of 21.05% as indicated by anaerobic culture and 16S rDNA PCR examination. More importantly, 7 samples were found to have visible bacteria upon histological examination and the bacteria were here considered* P. acnes*. We concluded that some nonpyogenic degenerated intervertebral discs had latent* P. acnes* infection, and the bacteria were observable via histology.

Up to now, direct histological reports of bacteria in nonpyogenic intervertebral discs have been rare. That may be the cause of the vigorous debates surrounding this topic. Stirling et al. were the first authors to report the presence of low-virulence anaerobic bacteria inside intervertebral discs, and they did so via Gram-staining, but the images were not classic or representative [7]. McLorinan et al. identified* P. acnes* using immunofluorescence microscopy, but they found that the bacteria colonized as single cells more than in large aggregates so the immune-labeled* P. acnes* was attributed as contamination [13]. Recently, Coscia et al. examined degenerated intervertebral discs with Gram-staining, but none of the samples had any visible microorganisms [2].

Here, 16S rDNA PCR served as a primary method of screening method due to its high sensitivity [18]. Histological observation was conducted because it was more specific and because it is considered the gold standard. In fact, 8 samples were found to be culture-positive but histologically negative but the reasons were complicated. One possibility is that the amount of* P. acnes* may be low in intervertebral discs and therefore it was not easy to observe them directly under histology. The study from Stirling et al. suggested that not very many bacteria were in the intervertebral discs [7]. However, another possibility was that these 8 samples may have suffered contamination. When a bacteria-free disc was contaminated by exogenous* P. acnes* during tissue harvesting, it would produce false-positive culture results of 16S rDNA PCR, but the histological results were still negative. In summary, it is difficult to determine why the 8 samples were culture-positive but histologically negative. More studies are needed to resolve this problem.

Contamination is the biggest reason to question the presence of* P. acnes* in nonpyogenic intervertebral discs. However, the histological evidence in this study indicated that not all of the isolated* P. acnes* were contaminated, and at least 7 samples were definitely original growth. First, if the bacteria came from contamination, they could be expected to remain on the surfaces of harvested tissues. In fact, tissue sectioning showed the bacteria to be present within the tissues rather than on the surface. Furthermore, the observed bacteria grew in clusters, further suggesting that they were original growth rather than contamination. Finally, cultures of the adjacent muscles in these 7 patients were negative, indicating that the incision was not contaminated by exogenous* P. acnes* during surgery and the harvested intervertebral discs had a low possibility of contamination. In this way, the observed bacteria could be considered original growth inside the intervertebral discs.

Previous studies have reported some cases of* P. acnes*-induced pyogenic discitis or spondylodiscitis. However, the patients included in this study did not have any signs or symptoms of discitis, and the reasons for this may be complicated. First, the amounts of invaded bacteria may be a key factor in different prognoses. In addition, the different infection pathway by which* P. acnes* invades intervertebral discs may lead to different clinical outcomes. Conventional viewpoints suggest that* P. acnes* invades intervertebral discs via iatrogenic operations, which may result in discitis, while other routine modes of infections lead to latent infection [5]. Finally, immunity of the patient is another key factor affecting clinical outcomes.

HE and Brown-Brenn staining were used to visualize the bacteria in this study. Although it is not a routine method of detecting bacteria, HE staining was able to indicate the presence of* P. acnes* or other microorganisms in tissues [19, 20]. Even the positive rates of HE staining were much higher than other staining methods in the detection of* Campylobacter pylori* in gastric antral biopsies [21]. Brown-Brenn staining is a modified form of Gram-staining used to stain bacteria in tissues directly [22]. It has some advantages over traditional Gram-staining, because the backgrounds created by traditional Gram-staining are so dark that it can be difficult to observe bacteria in tissue. In these ways, these two methods of staining may be more effective in the identification of bacteria in intervertebral discs than traditional staining.

Unfortunately, this study has some specific limitations. First, immunohistochemical labeling of* P. acnes* was not conducted in this research. However, due to the diverse phylogenetic formation of* P. acnes* in intervertebral discs [23], it can be difficult to label the bacteria with any unique antibody. In addition, other low-virulence anaerobic bacteria, such as* Coagulase-negative staphylococci*,* Corynebacterium propinquum*, and* Staphylococcus epidermidis*, were not examined in this study. A more comprehensive work should be performed to confirm the presence of other bacteria. Finally, more intervertebral samples should be included and investigated via histological observation to confirm the findings of this study.

## 5. Conclusion

*P. acnes* was absolutely confirmed to be present in some nonpyogenic degenerated intervertebral discs, at a prevalence of about 21.05%. This was here confirmed by histological examination. Latent infection of* P. acnes* may be crucial to many disc diseases, and targeting these bacteria may be a promising therapeutic method.

## Figures and Tables

**Table 1:** Prevalence of *P. acnes* identified with anaerobic culture and 16S rDNA PCR examination.

	Total numbers	Discs with* P. acnes*		Discs with unidentified bacteria		Discs without any bacteria
		Disc only	Disc and muscle both	Disc only	Disc and muscle both	
16S rDNA PCR	76	16	4	2	1	53
Gram-staining		Rod-like Gram-positive bacterium	Rod-like Gram-positive bacterium	N/A	N/A	

Positive rates		21.05%	5.26%	2.63%	1.32%	69.74%
